# Endocrine Activity of Extraembryonic Membranes Extends beyond Placental Amniotes

**DOI:** 10.1371/journal.pone.0005452

**Published:** 2009-05-08

**Authors:** Lori C. Albergotti, Heather J. Hamlin, Michael W. McCoy, Louis J. Guillette,

**Affiliations:** 1 Department of Biology, University of Florida, Gainesville, Florida, United States of America; 2 Department of Biology, Boston University, Boston, Massachusetts, United States of America; Pennsylvania State University, United States of America

## Abstract

**Background:**

During development, all amniotes (mammals, reptiles, and birds) form extraembryonic membranes, which regulate gas and water exchange, remove metabolic wastes, provide shock absorption, and transfer maternally derived nutrients. In viviparous (live-bearing) amniotes, both extraembryonic membranes and maternal uterine tissues contribute to the placenta, an endocrine organ that synthesizes, transports, and metabolizes hormones essential for development. Historically, endocrine properties of the placenta have been viewed as an innovation of placental amniotes. However, an endocrine role of extraembryonic membranes has not been investigated in oviparous (egg-laying) amniotes despite similarities in their basic structure, function, and shared evolutionary ancestry. In this study, we ask whether the oviparous chorioallantoic membrane (CAM) of chicken (*Gallus gallus*) has the capability to synthesize and receive signaling of progesterone, a major placental steroid hormone.

**Methodology/Principal Findings:**

We quantified mRNA expression of key steroidogenic enzymes involved in progesterone synthesis and found that 3β-hydroxysteroid dehydrogenase, which converts pregnenolone to progesterone exhibited a 464 fold increase in the CAM from day 8 to day 18 of embryonic development (F_5, 68_ = 89.282, p<0.0001). To further investigate progesterone synthesis, we performed explant culture and found that the CAM synthesizes progesterone *in vitro* in the presence of a steroid precursor. Finally, we quantified mRNA expression and performed protein immunolocalization of the progesterone receptor in the CAM.

**Conclusions/Significance:**

Collectively, our data indicate that the chick CAM is steroidogenic and has the capability to both synthesize progesterone and receive progesterone signaling. These findings represent a paradigm shift in evolutionary reproductive biology by suggesting that endocrine activity of extraembryonic membranes is not a novel characteristic of placental amniotes. Rather, we hypothesize that these membranes may share an additional unifying characteristic, steroidogenesis, across amniotes at large.

## Introduction

A key defining characteristic of amniotes (mammals, reptiles, and birds) is the formation of four extraembryonic membranes during embryonic development; the amnion, chorion, allantois, and yolk sac [1]. Fusion of the chorion and allantois forms either the chorioallantoic placenta in viviparous (live-bearing) species, or the chorioallantoic membrane (CAM) in oviparous (egg-laying) species [2]. Both the chorioallantoic placenta and CAM perform functions crucial for embryonic survival and development [1], [2]. Yet, only the placenta, which is a composite structure composed of extraembryonic membranes and maternal decidua, is classified as an endocrine organ [2], [3].

The mammalian chorioallantoic placenta synthesizes, transports, and metabolizes a suite of steroid and peptide hormones [2]–[4]. Of these, placental progesterone (P4), plays a key role in the maintenance of pregnancy [5], timing of birth [6], and promotes growth of the embryo [7] and placenta [8], [9]. Historically, endocrine properties of the placenta have been viewed as an innovation of eutherian mammals [2]. However, evidence of an endocrine placenta in three species of viviparous lizards [10], [11], [12] has recently called this traditional eutheriancentric view into question.

Examination of mammalian [3], [13], [14] and lizard [12] placentas has revealed that both extraembryonic membranes and maternal tissues contribute to hormone synthesis and metabolism. Therefore, we asked whether the extraembryonic membranes of oviparous amniotes could also play a role in the production of hormones during embryonic development. Although some differences do exist in the formation of the chorionic ectoderm between placental and oviparous amniotes [15], such an investigation is warranted given that the extraembryonic membranes share numerous similarities in their basic structure and function that are conserved across amniota. The presence of steroidogenic activity in the extraembryonic membranes of an oviparous amniote would imply a more ancient origin of endocrine function for these membranes than is currently believed.

Both the chorioallantoic placenta and CAM are derived from chorion and allantois [2], [15] Therefore, if the CAM has similar steroidogenic properties as the chorioallantoic placenta, then it should synthesize key placental hormones, such as P4. In this study we examined the potential activity of P4 in the CAM of chicken (*Gallus gallus*). To confirm P4 activity we must demonstrate that: (1) the oviparous CAM has the molecular mechanisms in place to perform steroidogenesis and synthesis of P4. Indeed, we show mRNA expression patterns of key steroidogenic enzymes involved in P4 biosynthesis. (2) The CAM is able to synthesize P4. We demonstrate that *in vitro* P4 synthesis takes place in the CAM and is not a product of steroids in the yolk or embryo, by isolating the CAM from other tissues and testing for synthesis directly in the presence of a steroid hormone precursor. (3) The CAM is capable of receiving the P4 signal through an appropriate receptor. Again, we demonstrate, via mRNA expression and protein immunolocalization of the progesterone receptor, that the CAM is capable of modulating P4 activity.

## Results

### The CAM has the required molecular mechanisms to perform steroidogenesis and synthesis of progesterone

**Figure 1:**
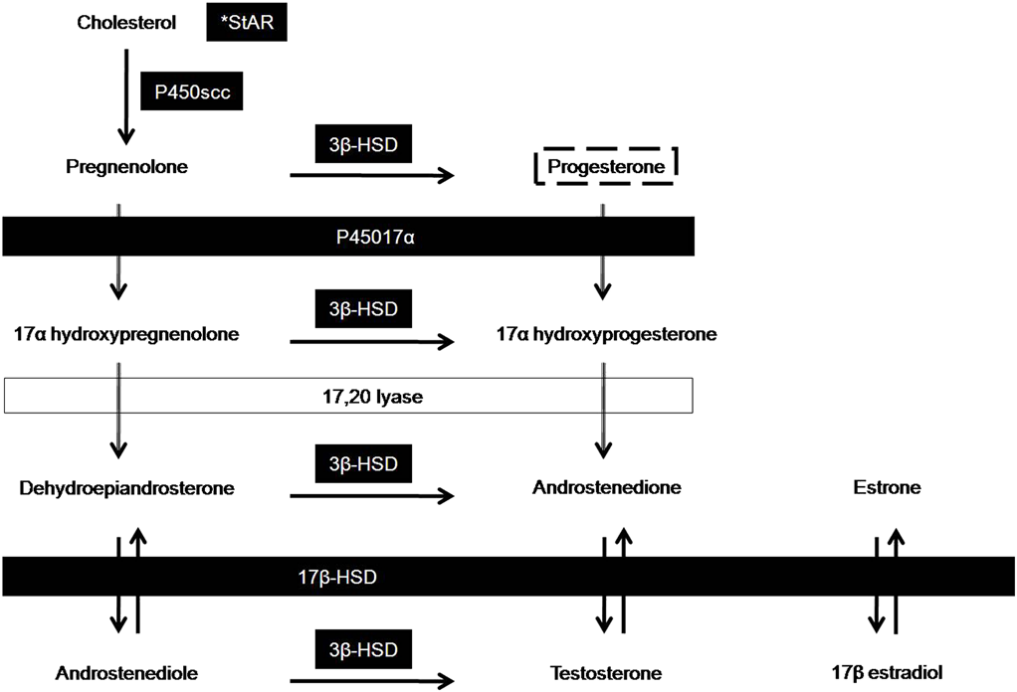
Steroid biosynthesis pathway. A simplified version of steroid biosynthesis highlighting the specific steroidogenic enzymes investigated in this study. Filled boxes highlight the steroidogenic enzymes examined by RT-qPCR. Progesterone (P4) is highlighted as the focus of this study. First, the transport protein, steroidogenic acute regulatory protein (StAR) is needed to facilitate the movement of cholesterol from the outer to inner mitochondrial membrane. Cholesterol is then converted to pregnenolone by the action of cytochrome side-chain cleaving enzyme (P450scc). Pregnenolone can then be converted to either 17α-hydroxypregnenolone by 17α-hydroxylase (P45017α) or to P4 by 3β-hydroxysteroid dehydrogenase (3β-HSD). P4 can either be a final product in this pathway or serve as a precursor in the synthesis of glucocorticoids, androgens, or estrogens. 17β-hydroxysteroid dehydrogenase (17β-HSD) functions in the conversion of weaker and stronger androgens and estrogens and was included in this study as a marker of upstream steroid enzyme activity [4], [16].

Cholesterol is required for the de novo synthesis of steroid hormones with steroidogenesis proceeding by the conversion of one steroid hormone to another by the action of specific enzymes [4]. To determine whether the oviparous CAM has the molecular mechanisms required for P4 synthesis, we examined mRNA expression of key steroidogenic enzymes in the steroid biosynthesis pathway [4], [16] (Figure 1). The relative levels and timing of mRNA expression were determined by harvesting CAM tissue, which forms on embryonic day 5, on embryonic days 8, 10, 12, 14, 16, and 18 and performing quantitative real-time RT-PCR (RT-qPCR) of mRNA coding for steroidogenic acute regulatory protein (StAR), cytochrome side-chain cleavage enzyme (P450scc), 17α-hydroxylase (P45017α), 3β-hydroxysteroid dehydrogenase (3β-HSD), and 17β-hydroxysteroid dehydrogenase (17β-HSD).

**Figure 2:**
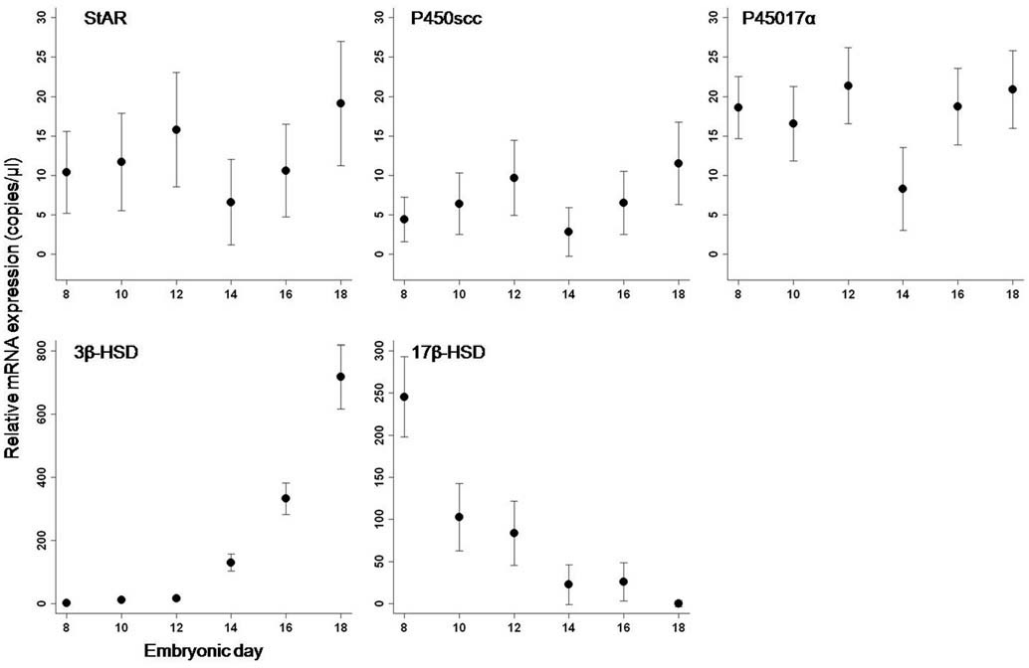
Relative mRNA expression of steroidogenic enzymes in the chick CAM. RT-qPCR analysis of mRNA coding for StAR, P450scc, P45017α, 3β-HSD and 17β-HSD on chick embryonic days 8 (n = 10), 10 (n = 17), 12 (n = 14), 14 (n = 10), 16 (n = 14), and 18 (n = 12). Data are reported as relative mRNA expression and represent mean normalized mRNA transcript number in copies/µL±SEM. 3β-HSD increased (F_5, 68_ = 89.282, p<0.0001) and 17β-HSD decreased (F_5, 68_ = 16.027, p<0.0001) significantly between embryonic day 8 and day 18.

We show the presence of steroidogenic enzyme mRNA in the CAM of an oviparous amniote (Figure 2). Overall, there was significant mRNA expression of StAR (F_1, 67_ = 65.222, p<0.0001), P450scc (F_1, 68_ = 58.489, p<0.0001), and P45017α (F_1, 66_ = 80.004, p<0.0001); however, the level of expression did not change significantly between embryonic day 8 and 18 for several components of the steroidogenic pathway (StAR F_5, 67_ = 1.144, p = 0.346), (P450scc F_5, 66_ = 1.618, p = 0.167), (P45017α F_5, 66_ = 1.787, p = 0.128). In contrast, we observed a 464 fold increase in 3β-HSD, which converts pregnenolone to P4, from day 8 to day 18 of development (F_5, 68_ = 89.282, p<0.0001). Detection of the steroidogenic enzymes required for the transport and conversion of cholesterol to P4 identifies a molecular mechanism for achieving P4 synthesis in the CAM. Additionally, the significant increase in 3β-HSD indicates that P4 synthesis in the CAM potentially increases through development similar to that of the chorioallantoic placenta through pregnancy [4], [17].

In contrast, 17β-HSD showed a significant decrease in expression, with mRNA expression decreasing to nearly zero by day 18 (F_5, 68_ = 16.027, p<0.0001), which could be associated with decreasing yolk androgens during development. Early in chick development, Elf and Fivizzani [18] reported high levels of androstenedione (A), testosterone (T), and dihydrotestosterone in the yolk, which decreased as development proceeds. They showed that yolk estradiol-17β remained constant through development until increasing on embryonic day 20, which is beyond the duration of our study. Because 17β-HSD is a key enzyme in the conversion between A and T [19] (Figure 1), high levels of yolk A and T earlier in embryonic development could explain why 17β-HSD mRNA expression in the CAM was initially high and then decreased through time. This scenario suggests that the CAM utilizes a maternal source of androgens during development. Taken together, these results indicate that the chick CAM has the molecular mechanisms in place to perform steroidogenesis in general and P4 synthesis in particular.

### The CAM is capable of *in vitro* progesterone synthesis

**Figure 3:**
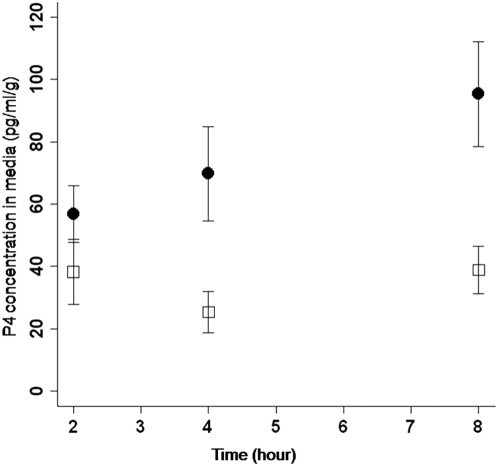
Progesterone synthesis in the chick CAM. CAM sections were incubated in culture media for 2, 4, or 8 hours either with (circles) or without (squares) cholesterol (plus cAMP) as a precursor. Concentration of P4 in the culture media is represented as pg/ml of P4 per g of CAM tissue (pg/ml/g). Addition of precursor significantly increased the concentration of P4 in the culture media (F_1, 58_ = 46.917 p<0.0001) with a significant interaction between time of incubation and addition of precursor (F_2, 58_ = 3.709, p = 0.0305). To determine background and cross-reactivity of the P4 assay, controls consisting of only cholesterol and cAMP were incubated for 8 hours with an average P4 concentration of 0.337 pg/ml/g±0.423 SEM (not shown).

Placental P4 synthesis in mammals is generally elevated from mid to late pregnancy [17]; therefore, to investigate P4 synthesis in the CAM, we harvested CAM tissue on embryonic day 18 and performed *in vitro* explant culture. Sections of CAM were incubated in culture media for 2, 4, or 8 hours either with or without cholesterol (plus cAMP) as a precursor. The concentration of P4 in the culture media was then quantified by radioimmunoassay. If the CAM is steroidogenic, addition of the steroid hormone precursor (cholesterol) to the culture media should stimulate increased P4 production. Indeed, our results showed a significant increase in concentration of P4 in the culture media following the addition of cholesterol precursor (F_1, 58_ = 46.917, p<0.0001) (Figure 3). Additionally, we observed a significant interaction between time of incubation and addition of precursor to the culture media (F_2, 58_ = 3.709, p = 0.0305). This result confirms that P4 synthesis can be induced in the chick CAM in the presence of a steroid hormone precursor. Further, we found that in the absence of precursor, the CAM produced a detectable level of P4 that did not change significantly during the assay (mean = 34 pg/ml/g±8.2 SEM, F_2, 21_ = 1.626, p = 0.2205) (Figure 3) suggesting that the CAM can exhibit endogenous P4 synthesis, but under the *in vitro* conditions used here this synthesis is likely limited by the lack of precursor. In contrast, a decrease in P4 concentration during the assay would have indicated that P4 detected at the first time point was perhaps the product of hormones leaching from this highly vascularized tissue. In sum, these results demonstrate that the chick CAM is capable of *in vitro* P4 synthesis. At present, we are unable to comment on the bioavailability of cholesterol in the CAM under *in vivo* conditions as these data do not currently exist.

### The CAM is capable of receiving P4 signaling through the progesterone receptor

**Figure 4:**
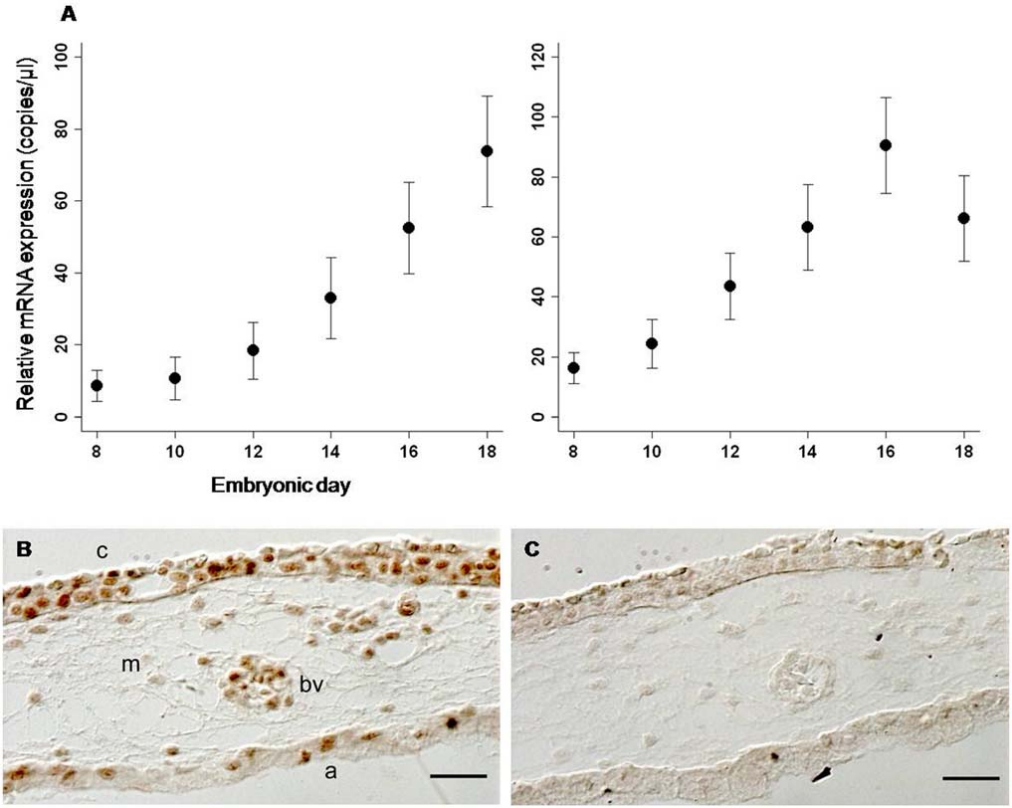
PR-ab and ERα mRNA expression and PR-ab immunolocalization. (A) RT-qPCR analysis of mRNA coding for PR-ab and ERα on chick embryonic days 8 (n = 10), 10 (n = 17), 12 (n = 14), 14 (n = 10), 16 (n = 14), and 18 (n = 12). Data are reported as relative mRNA expression and represent mean normalized mRNA transcript number in copies/µL±SEM. PR-ab (F_5, 68_ = 15.897, p<0.0001) and ERα (F_5, 66_ = 14.432, p<0.0001) increased significantly between embryonic day 8 and day 18. (B, C), PR-ab immunohistochemistry of embryonic day 18 CAM. (B) PR-ab positive section. Nuclear staining of PR-ab is localized predominately to the chorionic epithelium (c), and epithelial cells of blood vessels (bv). Positive nuclear staining is also present in allantoic epithelium (a), and mesenchyme (m). (C) Negative control of corresponding CAM section incubated without primary PR-ab antibody does not show specific nuclear staining. Scale bar represents 10 microns.

Finally, we examined the capability of the chick CAM to receive P4 signaling through an appropriate hormone receptor. As in human [20], chick P4 receptor (PR) is predominately expressed in two isoforms, PR-A and PR-B [21]. In chicken, the mRNA sequences of these isoforms are identical with the exception that PR-B has an additional 128 amino acids located at the N-terminus [21]. To identify both PR isoforms in the CAM, we designed primers that recognized the shared mRNA sequence (PR-ab) and performed RT-qPCR to examine relative expression during development. We show that PR-ab increased significantly through embryonic development (F_5, 68_ = 15.897, p<0.0001), demonstrating a 758% increase between embryonic day 8 and 18 (Figure 4A). We hypothesize that the observed increase in PR-ab expression in the CAM could be due to autoregulation by P4 and/or upregulation by an estrogen.

If CAM PR-ab is under autoregulation, i.e. P4 regulates its own synthesis, one might expect that as 3β-HSD and presumably P4 increases, a corresponding decrease in PR-ab expression would result [22]. However in placental tissues, P4 has been shown to maintain and possibly upregulate PR in rats [9], and to significantly increase the expression of PR in humans [23]. Therefore, it is possible that an increase in 3β-HSD and P4 could result in an increase in PR-ab expression in the CAM. If CAM PR-ab is upregulated by an estrogen, we might expect to see an upregulation of both chick PR [24] and estrogen receptor alpha (ERα) [25]. Therefore, we examined ERα mRNA expression in the CAM and found that ERα increased by 307% between embryonic day 8 and 18 (F_5, 66_ = 14.432, p<0.0001) (Fig. 4A) suggesting that an estrogen might play a role in PR regulation.

To determine if PR-ab mRNA is translated at the level of the protein, we performed immunolocalization of the nuclear PR with an antibody designed to recognize both chicken A and B isoforms [26]. We found PR-ab to be localized to the nucleus predominately in the chorionic epithelium and in the epithelial cells of mesenchymal blood vessels (Figure 4B). Positive nuclear staining was also found in the allantoic epithelia and mesenchyme. In total, mRNA expression and protein localization of PR-ab in the CAM indicate that this tissue can modulate the activity of P4 during embryonic development.

## Discussion

Collectively, our data indicate that the chick CAM is steroidogenic and has the capability to both synthesize progesterone and receive progesterone signaling. By demonstrating mRNA expression of steroidogenic enzymes, we show that the chick CAM has the molecular mechanisms in place to perform steroidogenesis and biosynthesis of P4. We demonstrate that the CAM is capable of *in vitro* synthesis of P4 in the presence of a steroid precursor. Additionally, we show that the CAM is capable of modulating P4 activity through the progesterone receptor.

In eutherian mammals, placental P4 plays a key role in the maintenance of pregnancy [5], timing of birth [6], and promotes growth of the embryo [7] and of the placenta itself [7]–[9]. Further, P4 has been observed to stimulate blood vessel proliferation [27] and maturation [28] in the mouse endometrium. Additionally, P4 has been suggested to play a role in human fetoplacental vascularization by regulating the proliferation of placental vascular smooth muscle cells [29] and through relaxation of placental blood vessels [30]. At present, we can only speculate on the role of P4 in the physiology of the CAM. We hypothesize that P4 in the oviparous CAM might be expected to serve similar roles as in eutherians contributing to the maintenance of embryonic development, timing of hatch, and growth of the embryo and of the CAM. Like the placenta, the CAM is a highly vascularized organ; therefore, we suggest that P4 might be involved in CAM blood vessel proliferation and maintenance.

Our findings represent a paradigm shift in evolutionary reproductive biology by indicating that steroidogenic activity of extraembryonic membranes is not a novel characteristic of placental amniotes. Further, we hypothesize that endocrine activity of extraembryonic membranes might be an evolutionarily conserved characteristic of amniotes. If steroidogenic activity of extraembryonic membranes is evolutionarily conserved, then the endocrine role of the amniote placenta is likely to have evolved initially in an oviparous ancestor.

This hypothesis that the extraembryonic membranes of amniotes are steroidogenic suggests an additional unifying characteristic of amniotes and has implications for evolutionary reproductive biology, particularly for the evolution of viviparity. It is currently estimated that within squamates (lizards and snakes) viviparity has independently evolved approximately 105 times [31]. In the transition from oviparity to viviparity in squamates, the period of time that eggs are retained within the uterus is increased and the thickness of the eggshell is decreased [32]. Eggshell reduction facilitates maternal-fetal gas exchange, but has also been proposed to function in the diffusion of chemical signals from the embryo to the mother in order to prolong gestation [32]. Thus, secretion of steroid hormones by the oviparous CAM could be important in establishing maternal recognition of pregnancy during the evolution of viviparity in this group. We suggest that evolutionary tinkering in the timing and spatial expression of steroidogenic genes in the CAM could lead to novel endocrine functions in communication with the maternal uterus; thus, facilitating the endocrine role of the chorioallantoic placenta.

## Materials and Methods

### CAM acquisition

Fertilized chicken (*Gallus gallus*) eggs were obtained from Charles River Laboratories (North Franklin, CT) and staged according to Hamburger and Hamilton [33]. CAMs were collected by removing the eggshell directly over the embryo and excising CAM away from embryo and yolk sac membrane. CAMs were washed in 1× phosphate buffered saline (PBS).

### RNA Isolation and reverse transcription

Dissected CAM was stored in the RNA preservative, RNA*later*® solution (Ambion) at 4°C. Total RNA was isolated from CAM with TRIzol® reagent (Invitrogen Life Technologies), purified with the SV Total RNA Isolation System (Promega), and reverse transcribed with the iScript™ cDNA Synthesis Kit (Bio-Rad). Concentrations and quality of RNA samples were evaluated by measuring optical density with a NanoDrop™ ND-1000 (Thermo Scientific) and by formaldehyde gel electrophoresis. Total RNA was treated with ribonuclease-free deoxyribonuclease I (DNase I; Qiagen) to remove any contamination of genomic DNA. 1 µg of total RNA was reverse transcribed and complementary DNA (cDNA) was diluted 10-fold and stored at −20°C until RT-qPCR analysis.

### Real-time quantitative polymerase chain reaction (RT-qPCR)

RT-qPCR analysis was performed on CAM samples from embryonic days 8 (n = 10), 10 (n = 17), 12 (n = 14), 14 (n = 10), 16 (n = 14), and 18 (n = 12). cDNA was analyzed in triplicate by RT-qPCR amplification using an iCycler MyIQ Single Color Real-Time PCR Detection System (Bio-Rad). Each 15-µL DNA amplification reaction contained a 10-fold dilution of 10× Gold Buffer (Applied Biosystems), 3 mM MgCl_2_, 200 µM dNTPs, 0.04% Tween-20, 0.4% glycerol, 1% DMSO, 500-fold dilution of SYBR Green (Invitrogen), 0.01 µM Fluorescein Calibration Dye (Bio-Rad), 0.2 µM of each primer, 0.67 µL of diluted cDNA, and 0.01 U AmpliTag Gold DNA polymerase (Applied Biosystems). RT-qPCR amplification conditions included an enzyme activation step of 95°C (10 min) followed by 40 cycles (reference genes) or 50 cycles (target genes) of 95 C (15 sec) and a primer specific combined annealing/extension temperature (1 min). The specificity of amplification was confirmed by melt-curve analysis. Triplicate data for each gene were averaged and amplification was determined by the absolute quantification method [34]. In brief, copy numbers were calculated from the cycle threshold (Ct) value by the linear regression of a standard curve. Standard curves for each target gene were generated from a plasmid containing the amplicon of interest. Controls lacking cDNA template were included on every RT-qPCR plate to determine the specificity of target cDNA. Additionally, to confirm that target cDNA was not contaminated by genomic DNA, RT-qPCR was performed with β-actin and PR-ab primers on the RNA isolated from every sample. To normalize mRNA expression levels, RT-qPCR was performed on all samples with five reference genes: β-actin, GADPH, ribosomal protein L8 (RPL8), RNA polymerase II polypeptide E (POLR2E), and ribosomal protein S13 (RPS13). The geometric mean was calculated according to geNORM [35] generating a normalization factor (NF) for each sample to correct for potential differences in RNA quality or quantity. For each target gene, absolute copy number was divided by the NF. Data are reported as relative mRNA expression and represent mean normalized mRNA transcript number in copies/µL±SEM.

### Cloning and sequencing of plasmids

RT-qPCR of pooled cDNA was used to generate a PCR product for each primer set. Amplified PCR products were separated on a 2% agarose gel and visualized by ethidium bromide on a Gel Doc EQ with Quantity One 4.6 software (Bio Rad). RT-qPCR products were purified by Wizard® SV Gel and PCR Clean-Up System (Promega) and purified samples were confirmed by electrophoresis on a 2% agarose gel. PCR products were cloned into a pGEM®-T Vector System (Promega). Plasmid DNA was purified using the Wizard® Plus SV Minipreps DNA Purification System (Promega) and sequenced on an ABI PRISM® 3100 Genetic Analyzer (Applied Biosystems) using a BigDye® Terminator v3.1 Cycle Sequencing Kits (Applied Biosystems). The specificity of cloned DNA was confirmed using BLAST against sequences available in Genbank. Clone DNA concentration was quantified by NanoDrop™ ND-1000, converted to copies/µl, and serially diluted in a solution containing 50 mM Tris-HCl (pH 8.3), 75 mM KCl, 3 mM MgCl2, and 5 µg/ml of tRNA.

### RT–qPCR primers

All Primers were designed to amplify mRNA-specific fragments from chicken coding sequences (NCBI) using Primer3 software [36] and were synthesized by Eurofins MWG Operon. Primer pairs were combined and diluted to a final concentration of 10 µM. Primer pairs are listed as forward (F), reverse (R), and the primer specific combined annealing/extension temperature used in RT-qPCR. RPL8-F 5′-CAA CCC CGA AAC AAA GAA AA-3′, R 5′-ATA CGA CCT CCA CCA GCA AC-3′ (62.4°C); β-actin-F 5′-TGC GTG ACA TCA AGG AGA AG-3′, R 5′-AGA GCT AGA GGC AGC TGT GG-3′ (60.9°C); POLR2E-F 5′-ATC AAC ATC ACG GAA CAC GA-3′, R 5′- GCA GCT CCG TCA CTT CTT CT-3′ (60°C); GADPH-F 5′-TAT CTT CCA GGA GCG TGA CC-3′,R 5′-TCT CCA TGG TGG TGA AGA CA-3′ (60°C); RPS13-F 5′- AAA GGC TTG ACT CCC TCA CA-3′, R 5′-ATG TTT GCG AAC AGC AAC AG-3′ (60°C); StAR-F 5′-GCC AAA GAC CAT CAT CAA CC-3′, R 5′-GAC CAA AGC ACT CAA CAG CA-3′ (61.6°C); P450scc-F 5′-GGT GTC TAC GAG AGC GTG AA-3′, R 5′-GTT GCG GTA GTC ACG GTA TG-3′ (64.4°C); P45017α-F 5′-GAC ATC TTC CCC TGG CTA CA-3′, R 5′-CAC AGT GTC CCC ACA GAA TG-3′ (64.4°C); 3β-HSD-F 5′-TCT CCA GGA AGG AGG CTT TA-3′, R 5′-GTA GAA CTG CCC CCT GAT GT-3′, (62.4°C); 17β-HSD-F 5′-GAG AGG GAC CAC GGT GCT GAT-3′, R 5′-AGT GGC GAA CAC TTT GAA CC-3′ (64.4°C); PR-ab-F 5′-CCC AGT CTC TAA CGC AAA GG-3′, R 5′-GCT CAA TGC CTC GTA AAA CA-3′ (65°C); ERα-F 5′-GAT AAT AGG CGC CAC AGC AT-3′, R 5′-TAG TCG TTG CAC ACA GCA CA-3′ (62.9°C).

### Sexing of embryos

To sex individuals used in RT-qPCR analysis, CAM genomic DNA was extracted from TRIzol® reagent (Zhu, Shirley, DNA extraction from TRIZOL organic phase, http://med.stanford.edu/labs/vanderijn/Protocols.html). DNA concentration was determined by Nanodrop and diluted to 100 ng/µL. Molecular sexing was performed as described in [37]. For *in vitro* tissue culture, day 18 individuals were sexed by visual inspection of embryonic gonads.

### *in vitro* explant culture

CAMs from 7 eggs were collected on embryonic day 18. CAMs were cut into 12 sections of approximately 0.1 g wet weight (mean = 0.104 g±0.0009 SEM) allowing for duplicate sections to undergo identical treatment regimes. CAM sections were incubated at 37°C on an orbital shaker in L-15 culture media (Invitrogen) either with or without cholesterol and cAMP as precursor. Precursor solutions and concentrations are based on King et al. 2004 [38]. For cholesterol, 22(R)-Hydroxycholesterol (Sigma) was dissolved in 95% ethanol (Fisher) to a final concentration of 10 µg/ml and combined with 1 mM Dibutyryl cAMP (Sigma). After 2, 4, or 8 hours of incubation, concentration of progesterone in the culture media was quantified by solid phase radioimmunoassay [39]. To determine background and cross-reactivity of the P4 assay, controls consisting of only cholesterol and cAMP were incubated for 8 hours with an average P4 concentration of 0.337 pg/ml/g±0.423 SEM.

### Immunohistochemistry and microscopy

Dissected CAM was fixed in 4% paraformaldehyde at 4°C overnight. Tissues were washed 3× in 1× PBS and stored in 75% ethanol at 4°C. CAMs were dehydrated, paraffin embedded, and sectioned at 8 microns. Tissue sections were deparaffinized in citrosolv and rehydrated through graded concentrations of ethanol to 0.1 M Tris buffered saline (TBS, pH 7.6). Immunohistochemistry was performed using the Vectastain® Universal Quick Kit, R.T.U. (Vector Laboratories) with the following modifications: for antigen retrieval, slides were autoclaved for 30 min in 10 mM sodium citrate buffer (pH 6.0). Sections were treated with 3% hydrogen peroxide for 20 min, blocked in normal horse serum (NHS) for 1 h, and treated with the Avidin Biotin blocking kit (Vector Laboratories). Between all incubation steps, slides were washed in TBS (5 min), 0.1 M TBS containing 0.2% Tween 20 (5 min), and again in TBS (5 min). CAM sections from day 16 (n = 5) and day 18 (n = 5) were incubated with a 1∶50 dilution of mouse monoclonal anti- progesterone receptor antibody (Ab-8), Thermo Scientific. PR Ab-8 recognizes both PR isoforms in chicken oviduct [26] and ovary [40]. Sections were treated with 3, 39-diaminobenzidine for 5 min (Vector Laboratories) and washed in running tap water for 5 min. A control section receiving normal horse serum in place of primary antibody was included on every slide. Slides were dehydrated, cleared and mounted with Permount™ mounting media (Fisher Scientific). Sections were imaged using a Leica DMRE microscope under DIC and Leica DFC 300 FX camera with Leica Firecam software.

### Statistical analysis

All statistical analyses were performed in the R statistical programming environment version 2.8.0 [41]. For gene expression analyses, the total numbers of mRNA transcripts for 5 control and 5 target genes from the CAM were determined by RT-qPCR. To quantify relative expression of target genes, we divided each sample by a normalization factor to yield normalized quantities (copies/µL). Normalization factors were estimated as the geometric mean expression of 5 control genes using geNORM [35]. Samples displaying a non-specific melt curve were excluded from the analysis and account for differences in number of samples between genes. To analyze each target gene, we used linear mixed effects models (LMMs) to estimate the parameters for relative mRNA expression over the 6 day experiment. For each analysis, embryonic day was treated as a fixed effect, and embryonic day, RT-qPCR plate, and sex of individuals were treated as random effects. Model assumptions were evaluated visually via examination of residuals and QQ plots and square-root transformations were performed when necessary to normalize errors (all genes were square root transformed except P45017α which did not require transformation). Outliers were identified from residuals and QQ plots and removed from the study (note that inclusion of outliers did not change patterns of significance, but were excluded from the final analysis because they have a disproportionate influence on mean estimates and caused violations of normality). The assumption of homogeneity of variances was met for all genes except 3β-HSD, which is likely due to the substantial changes in mean expression of 3β-HSD as the embryo developed. Thus for 3β-HSD variance was assumed to be a power of the estimated mean for each day and the exponent was estimated from the data as part of the estimation procedure [42]

For in vitro tissue culture, we used the same analytical approach described above using a LMM to estimate P4 concentration in culture media. In this analysis treatment (precursor versus control) and time (hour) are fixed effects on P4 concentrations (pg/ml/g of CAM tissue) and day of dissection, egg, replicate and sex of individual were treated as random effects. P4 concentration was square root transformed and two outliers, the largest value for control at 4 hours (206) and the largest value for precursor at 8 hours (213) were excluded from the analysis as outliers.
